# Real-Time Monitoring of Exosome Enveloped-AAV Spreading by Endomicroscopy Approach: A New Tool for Gene Delivery in the Brain

**DOI:** 10.1016/j.omtm.2019.06.005

**Published:** 2019-07-03

**Authors:** Nicola Salvatore Orefice, Benoît Souchet, Jérôme Braudeau, Sandro Alves, Françoise Piguet, Fanny Collaud, Giuseppe Ronzitti, Satoru Tada, Philippe Hantraye, Federico Mingozzi, Frédéric Ducongé, Nathalie Cartier

**Affiliations:** 1 INSERM UMR1169, Université Paris-Sud, Université Paris-Saclay, Orsay 94100, France; 2 CEA, Fundamental Research Division (DRF), Institut of Biology Francois Jacob, Molecular Imaging Research Center (MIRCen), Fontenay-aux-Roses 92265, France; 3 Neurodegenerative Diseases Laboratory, CNRS Laboratory of Neurodegenerative Diseases (UMR9199), Fontenay-aux-Roses 92265, France; 4 INTEGRARE, Genethon, INSERM, Université Evry, Université Paris-Saclay, Evry 91002, France; 5 Neurodegenerative Diseases Laboratory, CNRS CEA URA 2210, Fontenay-aux-Roses 92265, France

**Keywords:** adeno-associated virus, AAV6, AAV9, microvesicle, vexosome, endomicroscopy, gene therapy

## Abstract

Exosomes represent a strategy for optimizing the adeno-associated virus (AAV) toward the development of novel therapeutic options for neurodegenerative disorders. However, *in vivo* spreading of exosomes and AAVs after intracerebral administration is poorly understood. This study provides an assessment and comparison of the spreading into the brain of exosome-enveloped AAVs (exo-AAVs) or unassociated AAVs (std-AAVs) through *in vivo* optical imaging techniques like probe-based confocal laser endomicroscopy (pCLE) and *ex vivo* fluorescence microscopy. The std-AAV serotypes (AAV6 and AAV9) encoding the GFP were enveloped in exosomes and injected into the ipsilateral hippocampus. At 3 months post-injection, pCLE detected enhanced GFP expression of both exo-AAV serotypes in contralateral hemispheres compared to std-AAVs. Although sparse GFP-positive astrocytes were observed using exo-AAVs, our results show that the enhancement of the transgene expression resulting from exo-AAVs was largely restricted to neurons and oligodendrocytes. Our results suggest (1) the possibility of combining gene therapy with an endoscopic approach to enable tracking of exo-AAV spread, and (2) exo-AAVs allow for widespread, long-term gene expression in the CNS, supporting the use of exo-AAVs as an efficient gene delivery tool.

## Introduction

Upon fusion to an intermediate endocytic compartment, known as the multivesicular body, mammalian cells continuously secrete a large number of extracellular vesicles (EVs), known as exosomes, into the extracellular medium.^1^ These exosomes are capable of transferring lipid and genetic material with or without direct cell-to-cell contact,^2^, ^3^ mediating neuronal and glial cell communication in the CNS,^4^, ^5^, ^6^ and promoting neuronal repair, representing a novel system of intercellular communication.^7^ In the last decade, knowledge about the biological functions and potential therapeutic uses of exosomes has considerably increased. These microvesicles are involved not only in physiological functions (such as cell-to-cell communication) but also in disease pathogenesis, including autoimmune response,^8^, ^9^ neuronal dysfunction,^10^ and the development of neurodegenerative diseases.^11^, ^12^, ^13^ They have been also shown to be natural carriers of coding and noncoding RNA, including microRNA (miRNA), with the ability to induce *de novo* transcriptional and translational changes in the target cells.^14^

Considering the excellent safety profile of adeno-associated virus (AAV) vectors and their ability to transduce a large percentage of neurons and astrocytes in the CNS,^15^ the vulnerability to a host neutralizing antibody response as well as off-target liver uptake,^16^, ^17^, ^18^ accumulating evidence supports that AAV vectors enveloped in EVs, termed vexosomes (vector-exosomes), can be a promising gene delivery vehicle.^19^ The vexosomes led to an improvement of transduction in the cerebral tissue at a low dose, especially using AAV serotypes that have limited ability to cross the blood-barrier brain (BBB), and an increased resistance to human neutralizing antibodies compared to standard AAV vectors.^20^, ^21^, ^22^ Nevertheless, a more detailed understanding is still needed before exosome-enveloped AAVs (exo-AAVs) can become a suitable therapeutic carrier. In particular, the question of how and where the exo-AAVs are distributed in *vivo* after exogenous administration remains unclear.

Probe-based confocal laser endomicroscopy (pCLE), an imaging system for real-time fluorescence protein-based optical imaging of live animal cells,^23^ using proper dimensions and geometry can be used repeatedly over time in small animals to track individual cells *in vivo*. Thus, in this work, combining an imaging system and immunohistochemistry assay, we have detected and assessed the exo-AAV-mediated GFP spreading under the control of human phosphoglycerate kinase 1 (PGK1) compared to that mediated by unassociated AAV (std-AAV) vectors after unilateral intracerebral injection in the hippocampus of adult mice. This method of combining two different techniques for tracking and assessing gene expression in the hippocampus, a region that has emerged in the pathogenesis of numerous neurological diseases,^24^ has brought to light the following: (1) the pCLE system is a valid method to monitor the spreading of exo-AAV vectors in the brain; (2) exo-AAVs differ in their ability to spread in the brain contrary to std-AVV vectors; and (3) the exo-AAV vectors strongly enhance the ability of non-exo-AAV by providing a greater transfer of genes in neurons and oligodendrocytes, constituting a suitable cell-targeting strategy in specific neurological disorders.

## Results

### exo-AAVs Provide a Significant Brain Distribution

**Figure 1:**
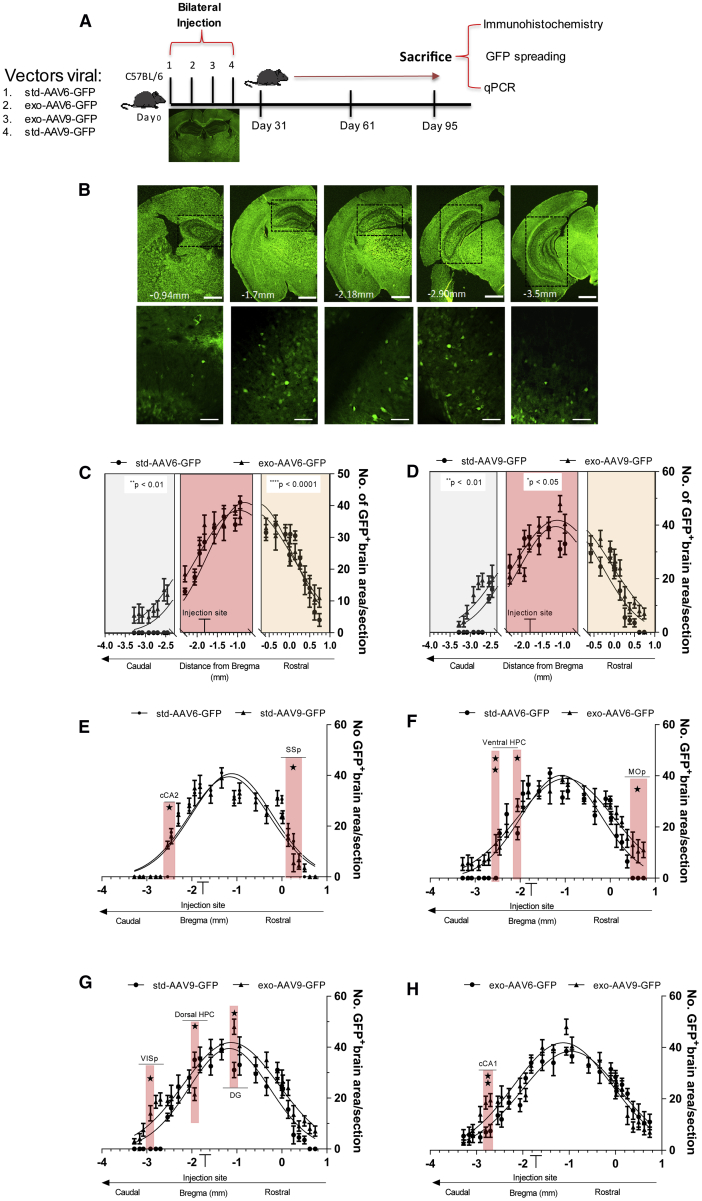
Spread of GFP-Expressing Exosome-Conjugated AAVs in the Brain (A) Experimental design of bilateral hippocampus stereotactic delivery of std-AAV6-9 and exo-AAV6-9 vectors (2 μL/injection site) expressing GFP into male C57BL/6J mice sacrificed at 95 days. (B) Coronal brain sections (100 μm)^57^ (n = 3–5 per group), with representative image of GFP^+^ cells (50 μm), were randomly chosen, scanned into the computer, and analyzed using a custom-designed Image-Pro-Macro. Fluorescence imaging in coronal brain slices was captured using a Leica DM600B at 5× magnification. (C and D) Gaussian distribution represents the spread within the brain of (C) std-AAV6-GFP (amplitude: 40.9) versus exo-AAV6-GFP (amplitude: 39.5) and (D) std-AAV9-GFP (amplitude 38.5) compared to exo-AAV9-GFP (amplitude: 41.8). (E and F) Sidak’s post hoc comparison revealed significant differences in (E) SSp (p = 0.0104) and cCA2 (p = 0.0261) between std-AAV6 and std-AAV9 and in (F) MOp (p = 0.0366) and ventral HPC (p = 0.0249; p = 0.0049) between std-AAV6 and exo-AAV6. (G and H) Significant difference in DG (p = 0.0116), dorsal HPC (p = 0.0153), and VISp (p = 0.0341) between std-AAV9 and exo-AAV9 and (H) in cCA1 (p = 0.0066) between exo-AAV6 and exo-AAV9. The x axis shows approximate distance from bregma (mm). cCA1, posterior hippocampus CA1; cCA2, posterior hippocampus CA2; DG, dentate gyrus; dorsal HPC, dorsal hippocampus; MOp, primary motor cortex; SSp, primary somatosensory cortex; ventral HPC, ventral hippocampus; VISp, primary visual cortex. Data are presented as mean ± SEM. *p < 0.05, **p < 0.01.

The first aim of this study was to compare the rostral-caudal distribution of viral vectors between exosome-conjugated AAVs and std-AAVs. To estimate their rostro-caudal spread following a single bilateral injection into the mouse hippocampus, we determined the spatial distribution of GFP expression in the brain for each viral vector. Comparing each sub-area close to the injection site (until 1 mm), a significant enhancement in GFP expression was observed in anterior-posterior coordinates by std-AAV6 compared to exo-AAV6 construction (Figure 1C), while a statistical difference between std-AAV9 and exo-AAV9 was observed in the GFP-positive brain areas close to the injection site and in the most caudal regions (Figure 1D).

At 3 months after injection, we observed that both std-AAV serotypes gained the best-fit value (std-AAV6, 40.9; and std-AAV9, 38.5) of GFP-positive brain areas surrounding the injection tract (Figure 1E); however, we identified in the primary somatosensory cortex (SSp) the brain region significantly more transduced by std-AAV6 while in the posterior hippocampus (cCA2) by std-AAV9. Although exo-AAV6 and exo-AAV9 showed a best-fit value (exo-AAV6, 39.51; exo-AAV9, 41.83) close to those observed with their respective std-AAV vectors, we identified in the primary motor cortex (MOp) and in the ventral hippocampus (ventral HPC) the brain regions significantly more transduced by exo-AAV6 (Figure 1F). The primary visual cortex (VISp) and dentate gyrus (DG) were the brain regions significantly more transduced by exo-AAV9 (Figure 1G), contrary to the dorsal hippocampus (HPC) that was more transduced by the std-AAV9 vector (Figure 1G).

### AAV6 and AAV9 Vector-Mediated Neuronal Tropism Can Be Enhanced in Association with Exosomes

**Figure 2:**
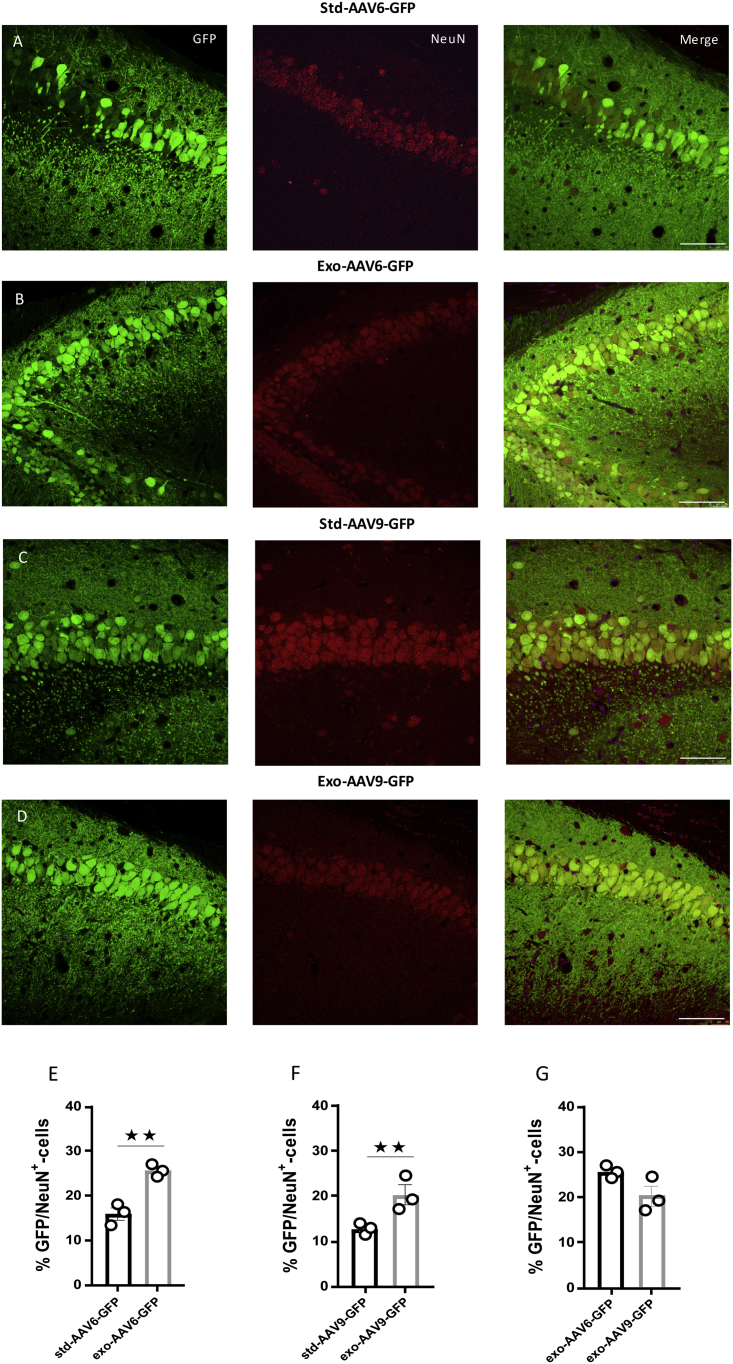
Neurons Are Preferentially Targeted by Exosome-Conjugated AAVs (A and B) Co-localization of GFP (green) with the neuronal marker NeuN (red) expressed by (A) std-AAV6 and (B) exo-AAV6 vectors. (C and D) Co-localization of GFP (green) with the neuronal marker NeuN (red) expressed by (C) std-AAV9 and (D) exo-AAV9 vectors. Slices were stained for neuronal marker NeuN at 3 months after bilateral injection into hippocampus. Images were taken using a Leica SP8 confocal microscope at 40× magnification. The merge image of both channels (GFP-NeuN) is shown on the right panel. Scale bar 20 μm. (E and F) Graphs represent GFP-NeuN-positive cells quantified and expressed as a percentage of total GFP-positive cells between (E) std-AAV6 and exo-AAV6 and (F) std-AAV9 and exo-AAV9. A preferential target neuronal was observed in exo-AAV6 and exo-AAV9 compared with std-AAV6 and std-AAV9 (n = 12; p = 0.0028; p = 0.0019). (G) No difference between exo-AAV6 and exo-AAV9 vectors. The data were analyzed by two-way ANOVA with Tukey post hoc test. Data are presented as mean ± SEM. **p < 0.01.

**Figure 3:**
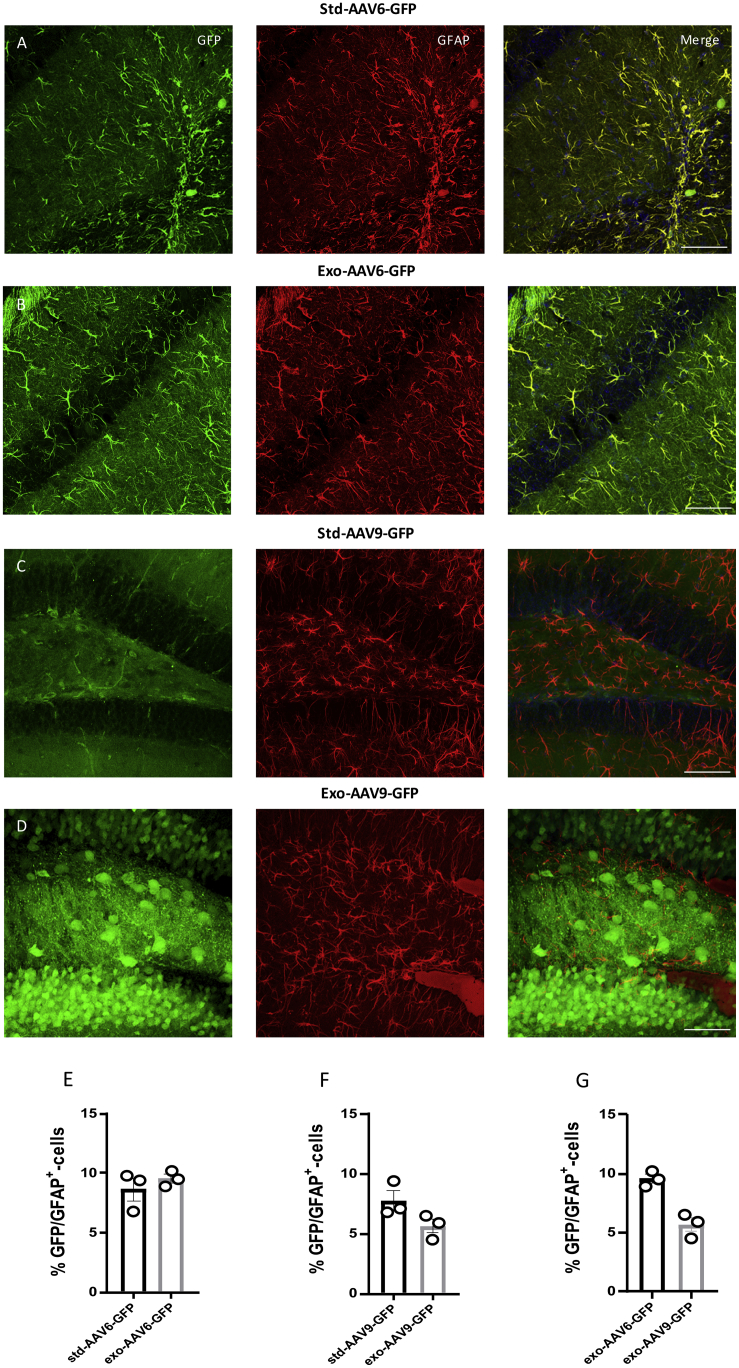
Conjugated Exosome AAV Is Not Sufficient to Mediate the Transduction of Astrocytes (A and B) Co-localization of GFP (green) with the astrocyte marker GFAP (red) expressed by (A) std-AAV6 and (B) exo-AAV6 vectors. (C and D) Co-localization of GFP (green) with GFAP (red) expressed by (C) std-AAV9 and (D) exo-AAV9 vectors. Slices were stained for astrocyte marker GFAP at 3 months after bilateral injection into the hippocampus. Images were captured by a Leica SP8 confocal microscope at 40× magnification. The merge image of both channels (GFP-GFAP) is on the right panel. Scale bar 20 μm. (E and F) Quantification of GFP-GFAP-positive cells expressed as a percentage of total GFP-positive cells between (E) std-AAV6 and exo-AAV6 and (F) std-AAV9 and exo-AAV9 (n = 12). (G) No significant difference between exo-AAV6 and exo-AAV9 vectors. The data were analyzed by two-way ANOVA with Tukey post hoc test. Data are presented as mean ± SEM.

Dissimilarities in the tropism efficiency are reported among the AAV serotypes across cell types; these problems limit their utility. Thus, it’s important to develop optimized vector design having a specific tropism for the desired target tissue. In our study, we assessed the exo-AAVs’ and std-AAVs’ tropism for neuronal and non-neuronal cells 3 months after hippocampus injection. An immunostaining analysis was performed using cell-specific marker antibodies for neuronal cell type and sub-types. Although the percentage of exo-AAV6- and exo-AAV9-mediated GFP-positive cells that co-localized with neuronal nuclei (NeuN) minimally varied between each hippocampal CA subfield regions and DG, we observed in exosome conjugation with AAV6 and AAV9 an increase in neuronal tropism (Figures 2B and 2D) compared with that observed in non-conjugated std-AAVs (Figures 2A and 2C). Contrarily, by coronal slices immunostained against astrocytes (glial fibrillary acidic protein [GFAP]), a low co-localization was observed in std-AAV6- and exo-AAV6-injected mice (Figures 3A and 3B) or in std-AAV9- and exo-AAV9-injected mice (Figures 3C and 3D).

**Figure 4:**
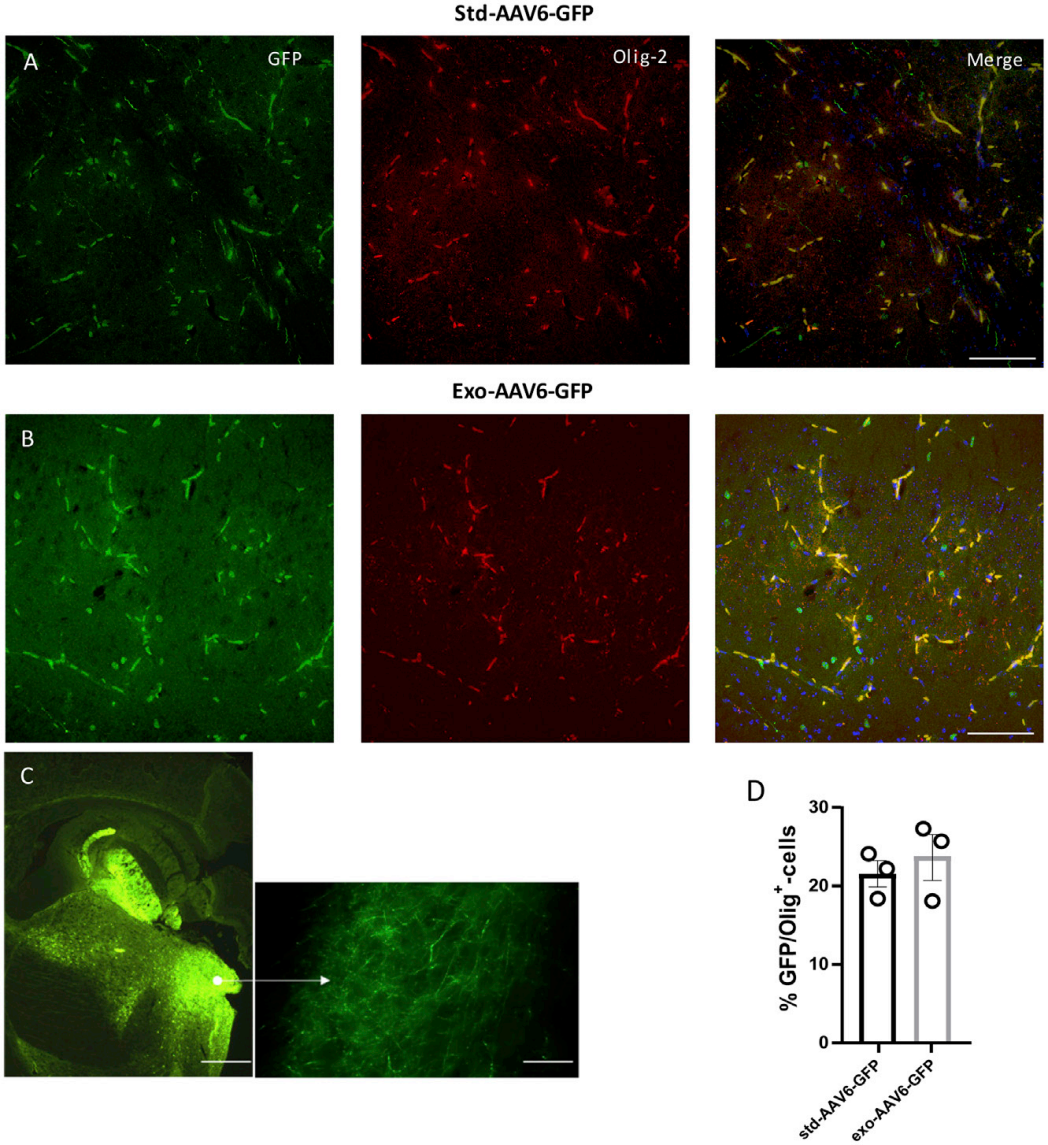
std-AAV6 and exo-AAV6 Mediate an Oligodendrocyte-Preferring Tropism in the Dorsal Striatum (A and B) Confocal imaging shows a high co-localization between GFP (green) and the oligodendrocyte marker (Olig-2-red) in the dorsal striatum (DS) expressed with (A) std-AAV6 and (B) exo-AAV6 at 95 days after bilateral injection into the hippocampus. Images were captured by a Leica SP8 confocal microscope at 40× magnification. The merge image showing both channels (GFP-Olig-2) is on the right panel. Scale bar 50 μm. (C) Representative image of GFP within the DS after stereotactic injection of std-AAV6 and exo-AAV6 vectors. Scale bar 100 μm. Fluorescence imaging was by a Leica DM600B. (D) Quantification of the GFP-Olig-2-positive oligodendrocytes transduced by std-AAV6 and exo-AAV6 expressed as percentages of co-localization between GFP and Olig-2 (n = 12). The data were analyzed by two-way ANOVA with Tukey post hoc test. Data are presented as mean ± SEM.

**Figure 5:**
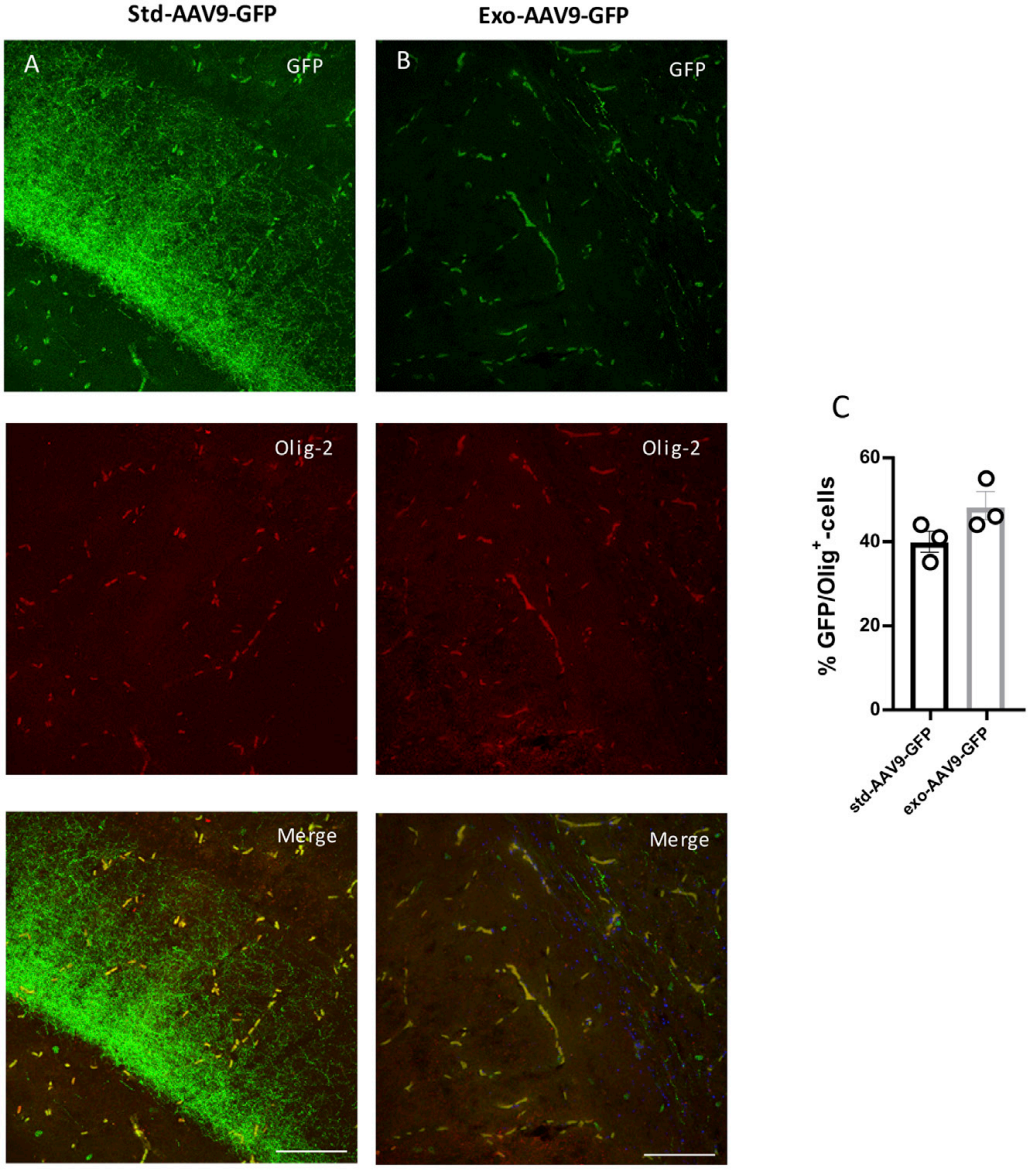
Oligodendrocyte Tropism of exo-AAV9 and std-AAV9 in the Granular Layer of the Hippocampus (A and B) Confocal imaging exhibits co-localization between GFP (green) and oligodendrocyte marker Olig-2 (red) after (A) std-AAV9 and (B) exo-AAV9 injection at 95 days. Images by a Leica SP8 confocal microscope at 40× magnification are shown. The combined image showing both channels (GFP-Olig-2) is on the right panel. Scale bar 20 μm. (C) Quantification of the GFP-positive oligodendrocytes transduced between std-AAV9 and exo-AAV9 expressed as percentages of co-localization between GFP and Olig-2 (n = 12). The data were analyzed by two-way ANOVA with Tukey post hoc test. Data are presented as mean ± SEM.

Interestingly, a population of GFP-positive cells lacking GFAP and NeuN expressions was detected after std-AAV6 and exo-AAV6 injection. These cells were lacking the morphological features that typically characterize astrocytes and neurons; their small size and morphology were consistent with oligodendrocyte cells. To identify the exact nature of these GFP-positive cells in different brain regions, we performed a double immunostaining study using anti-oligodendrocyte transcription factor (Olig-2), an oligodendroglial marker. Although we observed no GFP/Olig-2 co-localization in each hippocampal subfield region of interest expressing std-AAV6-GFP and exo-AAV6-GFP positivity (data not shown), the analysis of the dorsal striatum (Figure 4) displayed a spread of GFP expression in fibers. In particular, using either std-AAV6 (Figure 4A) or exo-AAV6 (Figure 4B), GFP-positive cells exhibited the typical morphology of striatal oligodendrocytes co-localizing with the oligodendrocyte marker Olig-2. In marked contrast, for mice receiving std-AAV9 and exo-AAV9, a co-localization between GFP and Olig-2 was mainly observed in the granular layer of the hippocampus (Figures 5A and 5B).

These results indicate that exosome conjugation with AAVs leads to an enhancement of gene expression principally in neurons and oligodendrocytes, with oligodendrocytes having differential expression in specific brain regions depending on the AAV serotype used. Furthermore, in 6 brain tissues of mice injected with exo-AAV6-9, the vector copy number was determined by qPCR in the cortex, hippocampus, and striatum. A low amount of vector copies was detected in the cortex for both viral vectors; on the contrary, although the difference was not statistically significant, the exo-AAV6-9-mediated expression of GFP in the hippocampus was confirmed by their respective amounts of vector copies (Figure S1A). Of note, an increase in the amount of vector copies in the striatum was detected in exo-AAV6-injected mice compared to that of exo-AAV9-injected mice, although no statistical difference was found.

### AAV6 and AAV9 Associated with Exosomes Increase the Spreading to Remote Brain Regions

**Figure 6:**
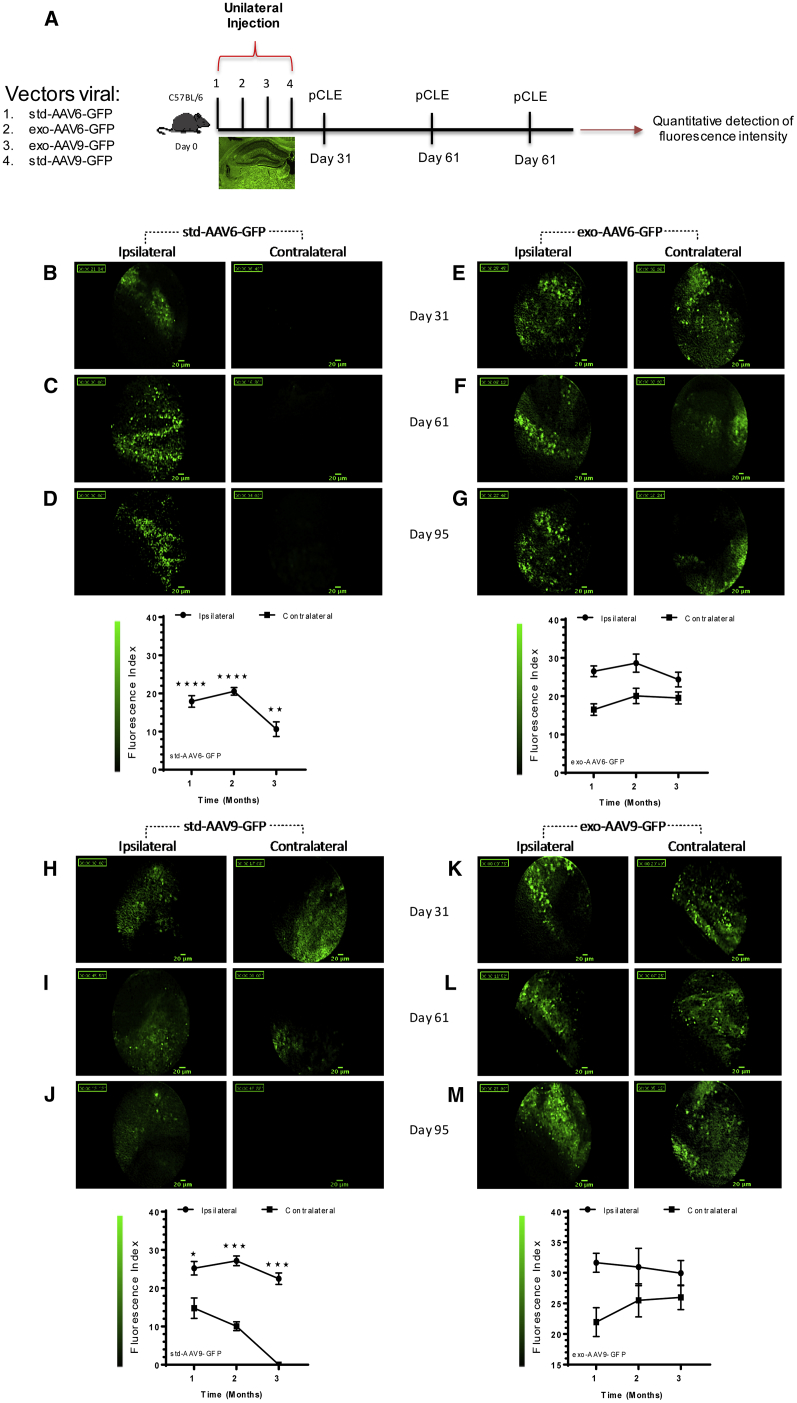
Detection of GFP Fluorescence via *In Vivo* Probe-Based Confocal Laser Endomicroscopy System (A) Schematic strategy in each group using stereotactic injection of std-AAV6-9 and exo-AAV6-9 vectors (2 μL) into the ipsilateral hemisphere and PBS (2 μL) into the contralateral hemisphere of adult C57BL/6 mice (n = 48) that underwent a confocal laser endomicroscopy system at 1, 2, and 3 months. Brain spreading of GFP fluorescent cells was detected using the confocal endoscope system (CellVizio-488) equipped with the S300 flexible microprobe containing 10,000 optical fibers that carry light from a continuous laser source at 488 nm to the living tissue. The GFP spreading in the contralateral hemisphere was monitored, introducing the flexible probe in the same small drill hole done on the skull in ipsilateral over viral vector injection sites of living mice using video data acquisition software (IC-Viewer, mkt). (B–G) GFP fluorescence brain spreading in the contralateral hemisphere at 1, 2, and 3 months from unilateral injection of (B–D) std-AAV6 with (E–G) exo-AAV6. (H–M) GFP fluorescence brain spreading in contralateral hemisphere at 1, 2, and 3 months from unilateral injection of (H–J) std-AAV9 with (K–M) exo-AAV9. Fluorescence pictures represent frames of 30- to 50-s acquired videos (scale bar 20 μm). Graphs represent the mean ± SEM between std-AAV6 compared to exo-AAV6 and std-AAV9 with exo-AAV9 of the relative fluorescence units (RFUs) detected over the time in the contralateral hemisphere by the 10,000 optical fibers per individual video frame. std-AAV6 (n = 8; interaction: F_2,6_ = 11.38, p = 0.0091; time: F_2,6_ = 11.38, p = 0.0091; spreading contralateral: F_1,6_ = 346.8, p < 0.0001. exo-AAV6 (n = 8; interaction: F_2,6_ = 1.078, p = 0.3982; time: F_2,6_ = 1.434, p = 0.3098; spreading contralateral: F_1,6_ = 27.53, p = 0.0019). std-AAV9 (n = 8; interaction: F_2,6_ = 7.064, p = 0.0265; time: F_2,6_ = 17.15, p = 0.0033; spreading contralateral: F_1,6_ = 161.8, p < 0.0001). exo-AAV9 (n = 8; interaction: F_2,6_ = 0.8112, p = 0.4877; time: F_2,6_ = 0.2112, p = 0.8154; spreading contralateral: F_1,6_ = 11.17, p = 0.0156). *p < 0.05, **p < 0.01, ***p < 0.001, ****p < 0.0001. Data are presented as mean ± SEM.

To assess if exo-AAVs are able to spread far from the injection site, we performed a unilateral injection experiment using each viral vector in the hippocampus. At specified time points (31, 61, and 95 days), viral vector diffusion was tracked using GFP detection by CellVizio-488 and Image-Cell software. The flexible microprobe was introduced into the brains of mice receiving either std-AAV6 (Figures 6B–6D) or its corresponding exosome (Figures 6E–6G). When compared at 95 days post-injection to the PBS-injected contralateral hemisphere, the GFP signal was significantly higher in the exo-AAV6- than in the std-AAV6-injected hemisphere. The pCLE system detected a weaker signal of std-AAV9-expressed GFP fluorescence in the PBS-injected contralateral hemisphere (Figures 6H and 6I) until 65 days compared to the signal of exo-AAV9-expressed GFP fluorescence in the PBS-injected contralateral hemisphere (Figures 6K and 6L), which was significantly higher. At 95 days from injection, no std-AAV9-mediated signal of GFP fluorescence was detected in the contralateral hemisphere (Figure 6J) in contrast to exo-AAV9 (Figure 6M). Finally, the exo-AAV9-mediated signal of GFP fluorescence was detected significantly more in contralateral hemispheres compared to exo-AAV6 at 95 days post-injection (Figure S2A).

**Figure 7:**
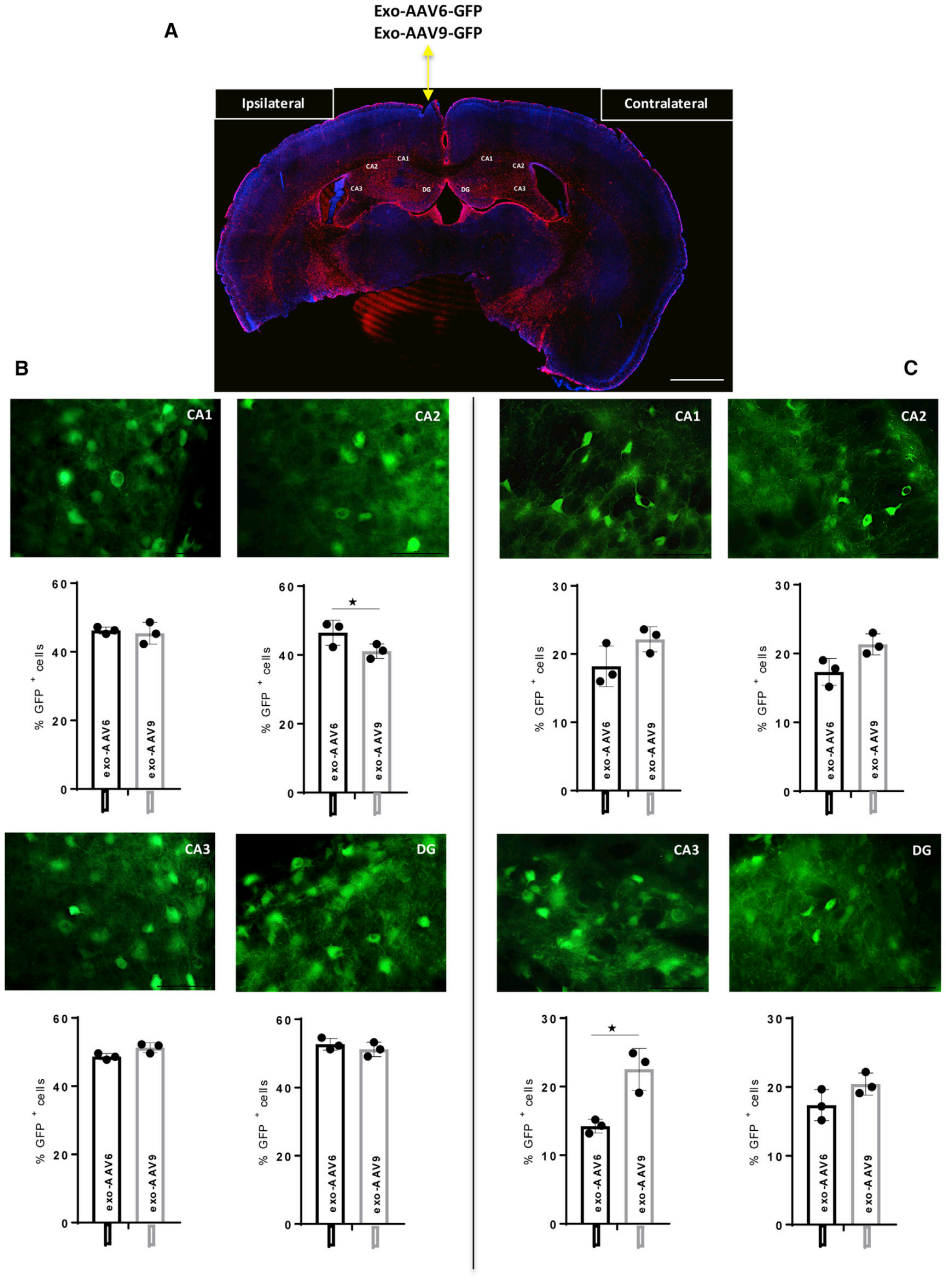
Quantification of exo-AAV6-9-Mediated GFP-Positive Cells in the Contralateral Hippocampus (A) Representative coronal brain section showing GFP distribution after unilateral injection of exo-AAV6-9 vectors (2 μL) into the ipsilateral hemisphere and PBS (2 μL) into the contralateral hemisphere of C57BL/6 mice. Images were captured by a Leica SP8 confocal microscope at 10× magnification. Scale bar 1 mm. (B) Quantification of GFP-positive cells visible enumerated in *Cornu Ammonis* subfields (CA1, CA2, and CA3) and in dentate gyrus (DG) in the ipsilateral hemisphere. CA2 (n = 3; p = 0.0319). (C) Quantification of GFP-positive cells visible enumerated in CA1, CA2, and CA3 and in DG in the contralateral hemisphere. CA3 (n = 3; p = 0.0397). Data were analyzed by an unpaired Student’s t with Mann-Whitney test and are expressed as percentages of GFP^+^ cells. Results were considered statistically significant with *p < 0.05 and levels of significance are indicated as follows: *p < 0.05. Data are presented as mean ± SEM. Images were by Leica DM6000 B fluorescence at 63× magnification. Scale bar 50 μm.

**Figure 8:**
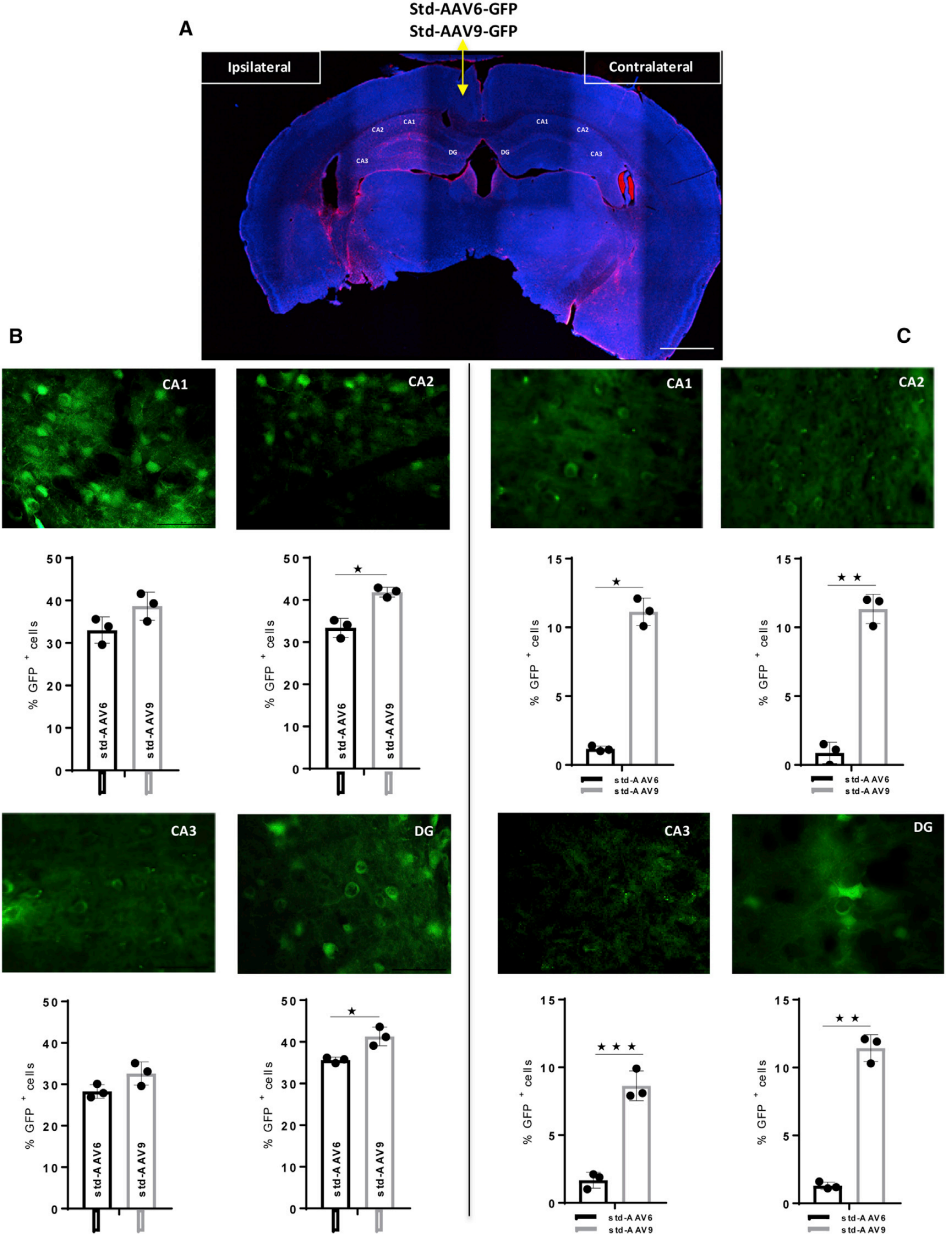
Quantification of std-AAV6-9-Mediated GFP-Positive Cells in the Contralateral Hippocampus (A) Representative coronal brain section showing GFP distribution after unilateral injection of std-AAV6-9 vectors (2 μL) into the ipsilateral hemisphere and PBS (2 μL) into the contralateral hemisphere of C57BL/6 mice. Images were captured by a Leica SP8 confocal microscope at 10× magnification. Scale bar 1 mm. (B) Quantification of GFP-positive cells visible enumerated in CA1, CA2, and CA3 and in DG in the ipsilateral hemisphere. CA2 (n = 3; p = 0.0236) and DG (n = 3; p = 0.0227). (C) Quantification of GFP-positive cells visible enumerated in CA1, CA2, and CA3 and in DG in the contralateral hemisphere. CA1 (n = 3; p = 0.0100), CA2 (n = 3; p = 0.0036), CA3 (n = 3; p = 0.0004), and DG (n = 3; p = 0.0029). Data were analyzed by an unpaired Student’s t with Mann-Whitney test and are expressed as percentages of GFP^+^ cells. Results were considered statistically significant with *p < 0.05 and levels of significance are indicated as follows: *p < 0.05, **p < 0.01, and ***p < 0.001. Data are presented as mean ± SEM. Images were by Leica DM6000 B fluorescence at 63× magnification. Scale bar 50 μm.

These results clearly indicate that AAV vectors have an enhanced ability to express GFP in the brain across a broader region when conjugated to exosomes. Moreover, an additional interesting aspect observed is the exosome AAV-mediated fluorescence from the injection site lasts until 95 days post-injection compared to 31 days using std-AAVs. To support these results, we counted the number of cells expressing GFP in *Cornu Ammonis* subregions (CA1, CA2, and CA3) and DG of the ipsilateral and contralateral hippocampus. In the CA2 subfield, at 95 days post-injection, the percentage of GFP-positive cells was significantly higher for the exo-AAV6 than the exo-AAV9 in the ipsilateral hemisphere (Figure 7B). Meanwhile, the observation done on the CA3 subfield of the contralateral hemisphere reported a statistically significant difference between both exo-AAVs (Figure 7C). Finally, a significant difference was observed between std-AAV6 and std-AAV9 in the CA2 subfield and DG of the ipsilateral hemisphere (Figure 8B). In each of the *Cornu Ammonis* subfields and DG of the contralateral hippocampus, the number of cells expressing GFP was significantly higher in std-AAV9 than in std-AAV6 (Figure 8C).

## Discussion

The role of gene delivery vectors is to transfer, preserve, and deliver a precise amount of genetic material of interest into target cells, achieving a sufficient level of transgene expression to achieve a desired therapeutic effect.^25^ Using non-neurotropic viral gene transfer vectors, such as lentivirus, adenovirus, and AAVs, several pre-clinical studies focused on CNS disorders have been conducted.^26^ This has opened the use of viral vectors in several clinical trials.^27^, ^28^ Although overcoming the systemic immune and inflammatory response^29^ and a limited transgene cargo capacity have made the viral vectors safer,^30^ efficient, and stable in gene delivery,^31^ a restricted spread in large brains is still a feature common to viral systems.^32^ Thus, to achieve a sufficient widespread distribution into other brain regions, a large volume of viral vector stock is required, with possible drawbacks in terms of manufacturing needs and risk of immune-mediated toxicities.

In this study, the use of two different AAV serotypes associated with exosomes represents a promising therapeutic strategy as gene vehicles capable of supporting long-term AAV spread throughout the brain, reaching regions far away from the injection site. This offers a major advantage both in reducing the number of injections required to achieve spread into a targeted brain region and to deliver the dose rate needed to achieve the target concentration, possibly decreasing the vector doses required for therapeutic efficacy. In addition, although no long-term studies have been performed to test the potential toxicity of exo-AAVs, our mice did not display any adverse reaction or behavioral changes after the intracranial surgery or during the subsequent period. It is well established that a major limitation of AAV-mediated gene transfer is the high prevalence of anti-AAV antibodies in humans,^33^, ^34^ which prevents their utilization in gene therapy trials. The implication of these results lends additional support to the hypothesis that the encapsulating exosome can shield AAV6 and AAV9 vectors from preexisting humoral immune response.

In line with this consideration, the first aspect observed using the exo-AAV6 and 9 was a greater GFP distribution from the injection site reaching the contralateral side, while with std-AAV6 and 9 it was localized mainly in the ipsilateral hemisphere. One possible explanation for this observed difference may be due to their capsid protein; enveloped AAV vectors cover their capsids with the EVs, allowing the viral genome to enter cells more easily. This is reflected by our ratios of rostral-caudal distance between std-AAVs and exo-AAVs (Figures 1C and 1D). Injections of exo-AAV6 and 9 resulted in the largest rostral-caudal spreads of GFP contrary to those of std-AAV6 and 9, which were relatively small. From here one question arises: if the largest rostral-caudal spread of GFP was found using exo-AAV rather than std-AAV vector, might the enhancement be linked to exo-AAV’s ability to efficiently escape the neutralizing antibodies? Additionally, there is a need for characterization of the exo-AAV preparations. It is unclear the extent to which membrane components could be contaminating the exo-AAV preparation. It is possible that the inclusion of membrane components in addition to DMEM could alter the tropism or the immune response after injection. While we have not presented evidence one way or the other on this issue, the authors would like to highlight this potentiality for the sake of authenticity. Future studies are essential to better define these points in large and small animal models.

Another key observation of the current study is the increase of transgene expression in neurons, without enhanced expression in astrocytes, using exo-AAV vectors under the PGK promoter. Application of viral vectors in gene therapy requires the choice of an optimal promoter able to direct the gene expression in specific target cells. Many promoters have been tested in the CNS, including cytomegalovirus (CMV), chicken beta-actin (CBA), and ubiquitous PGK1; although CMV and CBA are commonly used in gene transfer vectors, they have demonstrated potential drawbacks. Whereas *in vivo* CMV is prone to silencing over time after being transduced into the genome of cells in some tissues, such as hippocampus, striatum, and substantia nigra,^35^, ^36^, ^37^ CBA shows a significantly low expression in motor neurons (MNs).^38^ Previous investigations by Mellor et al.^39^ showed that when the PGK1 gene was cloned into a multicopy plasmid and expressed in yeast, Pgk1p accumulated to be as much as approximately 50% of total cell protein. Furthermore, different powerful expression vectors were constructed, based on the promoter region of the PGK1 gene, and these vectors have been used to study the expression of a number of heterologous genes.^40^, ^41^, ^42^ Although the activity of promoters can vary depending on host cells and experimental settings, based on the evidence, we chose the PGK1 promoter as the basis for construction of a new vector containing microvesicles.

The brain is an organ where many of the most devastating human neurological disorder processes arise, for which the causes remain elusive and treatments are still lacking. These diseases encompass a broad spectrum of pathological states and can have specific effects on the metabolism, development, and function of neurons. Several studies report a transduction efficiency of neurons and non-neuronal cells such as astrocytes using std-AAV6 and std-AAV9;^43^, ^44^, ^45^, ^46^ opposite to what we expected, we observed a brain region-specific tropism of neurons and oligodendrocytes using std-AAV6 and std-AAV9 conjugated with exosomes, while a low transduction in astrocytes was observed using both exo-AAV and std-AAV constructions. We wondered what could be the causes of these dissimilarities, considering two key factors: (1) the brain injection was performed using the same viral preparations and the same volume of injection, and (2) all viral vectors used in this study were made under the control of the same PGK promoter.

A possible reason might be the different cytoarchitectures among brain regions such as the cortex, hippocampus, and striatum that may influence the efficiency of exo-AAV and std-AAV-mediated gene delivery.^47^ Additionally, differences in viral vector manufacturing, purification, and route of administration have been previously documented to drive different cell tropism of AAV vectors in the brain.^48^ However, these results established that a modified AAV vector carrying an envelope exhibited a preferential *in vivo* tropism for neurons and oligodendrocytes in specific brain regions, representing a major departure from the normal neuronal tropism indicated by other AAV serotypes with dominant neuronal tropism after direct injection into the brains of rodents.^43^, ^49^, ^50^ In particular, several studies have described the ability of specific std-AAV serotypes to transduce oligodendrocytes at a low efficiency, requiring the use of oligodendrocyte-specific promoters to prevent expression in neurons, the preferred cell type for these vectors.^51^, ^52^ In addition, we hypothesize that, because AAVs enter target cell receptor-mediated endocytosis,^53^, ^54^ the exosome association may confer to the viral vector a new tropism via binding to different cell receptors.

Our study suggests that exo-AAVs might be applied to specifically transfect neurons and oligodendrocytes, constituting a tool of choice in gene therapy for neurological diseases, such as multiple sclerosis (MS), spinal muscular atrophy (SMA), or amyotrophic lateral sclerosis (ALS), where currently no effective curative therapy exists. Applying an *in vivo* imaging technology, with image resolution equivalent to confocal microscopy on living animals, it has been possible to track and detect the exo-AAV-mediated GFP expression throughout the brain. Conventional optics and scanners are large and bulky; in addition, they require wide surgical exposure that may lead to significant trauma. Confocal endomicroscopy is a minimally invasive method that has sufficient resolution and allows for model animals to be studied longitudinally, reducing the numbers needed per experiment and improving statistical power. This may allow for testing to see if an exo-AAV construct is capable of delivering the gene into a specific area of the CNS as a pre-translational screening. The current formulation for drug therapeutics fails to provide drug delivery into the correct brain regions; an efficient system of delivery is direly needed.

In conclusion, exo-AAV vector is a powerful tool to enhance vector spreading in the brain, and it might offer the potential to address significant unmet clinical needs.

## Materials and Methods

### Animals

The 8-week-old male C57BL/6J mice were purchased from Janvier (Le Genest-Saint-Isle, France). Mice were housed in a temperature-controlled room and maintained on a 12-h light-dark cycle. Mice were maintained under specific pathogen-free conditions. Food and water were available *ad libitum.* All procedures were approved by the Regional Ethics Committee in Animal Experiment No. 44 of the Ile-de-France region. All animal experiments were carried out in full accordance with the European Community Council directive (86/609/EEC) for the care and use of laboratory animals.

### Standard AAV and Exosome AAV Production

Standard AAV6-PGK-GFP and AAV9-PGK-GFP and their exo-AAV counterparts used in this study were produced using a slight modification of the adenovirus-free transient transfection methods.^55^ Briefly, adherent HEK293 cells grown in exo-free medium were transfected with the three plasmids containing the adenovirus helper proteins, the AAV Rep and Cap genes, and the inverted terminal repeat (ITR)-flanked transgene expression cassette. At 72 h after transfection, supernatant was harvested, and exo-AAV vectors were isolated by differential centrifugation, as described by György et al.^21^ From the same production, standard AAV vectors were purified from cells after lysing by sonication and treatment with Benzonase (Merck-Millipore, Darmstadt, Germany). Standard AAV vectors were then purified using two successive ultracentrifugation rounds in cesium chloride density gradients, as reported by Ayuso et al.^56^

After the first centrifugation (104,000 *g*, 24 h, 20°C), visible bands corresponding to AAV particles were then collected with a 10-mL syringe; under this condition, the vector particle bands were insufficiently separated to allow their reliable separation, and the bands were collected together with a syringe. Vector particles recovered from the first ultracentrifugation were then pooled and subjected to isopycnic separation by a second ultracentrifugation step (182,000 *g*, 24 h, 20°C), allowing a high-resolution separation of the AAV particles and facilitating precise visual recovery of the lower band corresponding to the genome-containing AAV particles. Exo-AAV vectors were isolated from the media 3 days after transfection using differential centrifugation as described earlier.^21^ Cells were depleted at 300 × *g* for 5 min and 1,000 × *g* for 10 min. Next, larger EVs (apoptotic bodies and microvesicles) were removed by a 20,000 × *g* spin for 60 min. The supernatant from the 20,000 × *g* spin was subjected to 100,000 × *g* centrifugation for 1.5 h. The exosome pellet was re-suspended in serum-free, antibiotic-free DMEM. Exo-AAV and AAV final products were respectively formulated in sterile PBS and PBS containing 0.001% pluronic (Sigma-Aldrich, St. Louis, MO) and stored at −80°C. Titers of AAV and exo-AAV vector stocks were determined using quantitative real-time PCR. Viral DNA was extracted using the viral NA small volume kit (Roche, Indianapolis, IN), according to the manufacturer’s instructions. The qPCR was performed in ABI PRISM 7900 HT Sequence Detector using Absolute ROX mix (Taqman, Thermo Fisher Scientific, Waltham, MA).

### Stereotactic Injections of exo-AAV

Mice were anesthetized by intraperitoneal (i.p.) injection of ketamine/xylazine (0.1/0.05 g/kg body weight) and mounted on a stereotactic device. A subset of mice (n = 44) received a unilateral intrahippocampal injection of exo-AAV or std-AAV vector at the dose of 1.5 × 10^11^ vector genomes (vg)/mL (2 μL/injection/site). As a control, 2 μL PBS solution was injected into the contralateral hippocampal hemisphere. After 1, 2, and 3 months from injection, the mice underwent a pCLE system that was applied to detect the GFP fluorescence in both hemispheres. A subset of mice (n = 12 for each group) that underwent molecular and immunohistochemical analyses received a bilateral intrahippocampal injection of both exosome-enveloped or std-AAV serotypes at the dose of 2 × 10^9^ vg/μL in 2 μL at the rate of 0.2 μL/min. All viral vectors were diluted in PBS 0.1 M. Stereotactic coordinates of injection sites from bregma were as follows: anteroposterior −2 mm, mediolateral ±1 mm, dorsoventral −2.25 mm and anteroposterior −2 mm, mediolateral ±1 mm, and dorsoventral −1.75 mm.

### *In Vivo* Laser Endomicroscopy Analysis

The mice underwent laser endomicroscopy analysis at different time points, at 1, 2, and 3 months after unilateral injection. Video data acquisitions and analyses were performed with a CellVizio-488 system and the Image-Cell software (Mauna Kea Technologies, France). Before video acquisition, the S300 microprobe was calibrated with a fluorescence calibration kit (Mauna Kea Technologies, France) to homogenize individual fiber signals by addressing the same value of background and giving the same potential for quantitative detection of fluorescence intensity to all fibers. Image cell acquisition software addresses colors and relative fluorescence units (RFUs) according to values of signal intensity, without affecting intrinsic data.

Positive fluorescence signal was set over 60. Cellular imaging was performed using a flexible microprobe containing 10,000 optical fibers that carry light from a continuous laser source at 488 nm to the living tissue. Fluorescence emitted by excitation of the GFP protein is carried back by optical fibers to the apparatus, where a dedicated set of algorithms reconstructs images in real time. The rate of acquisition is 12 frames/s, and the field of view and spatial resolution depend on the flexible probe used. With the S-300 flexible microprobe used here, the field of the reconstituted image covered a 300-μm circular diameter in a focal plane, 0–15 μm away from the probes optical window with a spatial resolution in the plane of 3.3 μm. This spatial resolution is rather low but compatible with the size of single cells (8–10 μm), meaning that individual cells could be observed even if they would appear blurred. The flexible probe was entered in the same small drill hole that was done in the skull over virus vector injection site and was moved slowly (0.3–0.6 mm/s). The choice to remount the mice on stereotactic has been intended to be sure that the probe was introduced in the same small drill hole that was used for the viral vector injection. Through this route, the probe was fixed to the vertical shaft of stereotaxic, introduced and moved slowly in the ipsi- and contralateral hemispheres.

### Tissue Preparation

C57BL/6J mice received an overdose of sodium pentobarbital and were sacrificed after a given time point (1, 2, and 3 months) following the unilateral/bilateral injection of viral vector systems. For biochemical analysis, mice were transcardially perfused with 0.1 M PBS solution; their hippocampus was dissected and quickly dropped in liquid nitrogen for storage at −80° until use. For immunohistochemistry analysis, in a subset of animals the PBS perfusion was followed by 4% paraformaldehyde (PFA). The brains were removed and post-fixed again in 4% PFA in 0.1 M PBS for 72 h and after transferred into sucrose gradient (15% and 30%). Coronal sections (50 μm) were obtained using the vibrating-blade microtome (Leica VT-100S) and collected serially.

### Immunohistochemistry and Image Acquisition

The immunohistochemistry was performed by blocking the coronal slices in PBS/0.1% Triton X-100 containing 5% normal goat serum (NGS, Gibco) at 4°C overnight. Subsequently, slices were washed three times for 10 min in PBS solution and incubated overnight at 4°C with primary antibodies: anti-GFAP (Dako) used to visualize astrocytes, anti-NeuN (Millipore) as neuronal marker, and Olig-2 (Millipore) to target oligodendrocytes. All primary antibodies were in PBS containing 5% NGS and 0.1% Triton X-100 at 4°C overnight. On the next day, after three washings for 10 min, the slices were incubated with the corresponding fluorescent secondary Alexa Fluor-conjugated antibodies (Thermo Fisher Scientific) in PBS containing 5% NGS and 0.1% Triton X-100 at 4°C overnight. Next, the slices were washed three times for 10 min with PBS, incubated for 30 min in DAPI solution, and mounted on superfrost slides (Thermo Scientific).

Coronal brain slices (50 μm) were acquired using a Leica SP8 confocal system equipped with a broadband white-light laser with an oil immersion objective 40×, 1.3 numerical aperture (NA). The excitation laser lines were set up to 488 nm for GFP and 633 nm for Alexa Fluor 633, both at 3% of intensity. Alternative scan mode imaging was used setting up the normal photodetector to 499–520 nm for GFP and the hybrid detector (HyD) photodetector to 644–702 nm for Alexa Fluor detection. In all cases, the white laser power was settled to 70%, the acquisition speed was 600 Hz, and the image resulted from the average of 4–6 laser scans. The Z-step size was settled to 3 μm. Deconvolution processes were done using Autoquant-X (Media Cybernetics, USA), through the blind adaptive point spread function (PSF) method with 10 iterations and low noise level. The images presented are the result of the maximum projection of the entire stack.

### Quantification of GFP-Positive Area and Transduction Cell Type

The 25 coronal brain sections (20 μm) between each section were picked for each of the viral vectors bilaterally injected into the hippocampus. Sections were aligned according to the distance between sections and anatomical landmarks based on the mouse brain.^57^ The first section was from ∼1.4 mm rostral to bregma, and the last section was ∼4.3 mm caudal to bregma, so the total rostral-to-caudal distance analyzed was ∼5.7 mm. Sections were scanned into the computer using a Microtek ArtixScan 4000tf (Microtek, Carson, CA) scanner at 4,000-dots/in resolution. The images were then analyzed with a custom-designed macro using the ImageJ software (NIH). The number of pixels in the section reaching the threshold was counted and converted to percent of area by using the total number of pixels for the whole section as the denominator of the GFP-positive area.

To calculate only the hippocampal coverage of GFP expression, the ImageJ software (NIH) was employed. The GFP coverage was calculated subdividing into three identical width regions the area (mm^2^) of GFP signal by the area of the targeted region (CA1, CA2, CA3, and DG for each animal), which were calculated separately and expressed as percentages of GFP coverage. To identify the transduction differences between exo-AAVs and std-AAVs, the coronal brain slides were digitized, and the hippocampal volume was delineated and quantified using a computer-based image analysis system (MCID software, Inter-Focus, Mering, Germany). To determine the proportion of GFP/NeuN^+^-GFAP^+^-Olig^+^ cells, slices were randomly chosen and quantified, first in the red channel to determine the number of NeuN^+^, GFAP^+^, and Olig cells under 40× objective, and combing red and green channels (GFP) to determine the number of co-stained cells. By obtaining the number for each type of cell, the percentage for each section was determined for GFP^+^/NeuN^+^, GFAP^+^, Olig-2^+^ to total NeuN^+^, Olig-2^+^, and GFAP^+^ cells. Only cells with DAPI-positive nuclei within the counting frames were considered.

### qPCR

Exosome AAV GFP vector genome copy numbers were measured by qPCR. Vector genome copy numbers were measured by qPCR in the cortex, striatum, and hippocampus using the Light Cycler 480 II (Roche, France) and the Light Cycler 480 SYBR Green I Master (Roche, France). Vector sequences (GFP) and mouse genomic *Adck3* (internal control) sequences were simultaneously amplified. The results (vector genome copy number per cell, VGC) were expressed as n-fold differences in the transgene sequence copy number relative to the *Adck3* gene copy (number of viral genome copy for 2N genome). Samples were considered vector negative if transgene sequence Ct value was >35. Each sample was analyzed in duplicate. At least three mice were scored per condition and results were expressed as mean ± SEM.

### Statistical Analysis

All graphs and statistical analyses were carried out with GraphPad Prism 7 software (La Jolla, CA). Gaussian function was applied to describe the distribution into the brain of viral vectors. A two-way ANOVA followed by Sidak’s post hoc test were performed to define the brain area significantly more transduced by exo-AAVs. To test the differences in transduction efficiency and of cell type tropism for the different viral constructions used, a two-way ANOVA (main factors: AAV carrier and time) was performed followed by a Tukey post hoc test. When analyzing the percentage of GFP-labeled cells per millimeter^2^ in *Cornu Ammonis* subregions (CA1, CA2, and CA3) and the DG of the hippocampus in both hemispheres from the different viral constructions, separate Student’s t test was applied followed by a Mann-Whitney test. Two-way ANOVA was also used to assess statistically significant differences of RFUs. Statistical significance was considered at p ≤ 0.05. Data were expressed using mean ± SEM.

## Author Contributions

N.S.O. carried out the design of the study and wrote the manuscript. B.S., S.A., F.P., and S.T. participated in the molecular and immunohistochemistry analyses and helped to draft the manuscript. J.B. participated in the statistical analyses and helped to draft the manuscript. F.C. and G.R. performed the viral vector production. F.M. performed the viral vector production and helped to draft the mansucript. F.D. participated in *in vivo* imaging analysis and helped to draft the manuscript. P.H. and N.C. read the manuscript. All authors read and approved the final manuscript.

## Conflicts of Interest

F.M. is an employee of Spark Therapeutics Inc.

## Supplemental Information

Document S1. Figures S1–S4

Document S2. Videos S1–S4

Document S3. Article plus Supplemental Information
