# Supplementary material for: Real-Time Monitoring of Exosome Enveloped-AAV Spreading by Endomicroscopy Approach: A New Tool for Gene Delivery in the Brain
Source: Mol Ther Methods Clin Dev. 2019 Jul 3;14:237–51. doi: 10.1016/j.omtm.2019.06.005 (PMC6699252; doi:10.1016/j.omtm.2019.06.005)

OMTM, Volume *14*

Supplemental Information

Real-Time Monitoring of Exosome Enveloped-AAV Spreading by Endomicroscopy Approach: A New Tool for Gene Delivery in the Brain

Nicola Salvatore Orefice, Benoît Souchet, Jérôme Braudeau, Sandro Alves, Françoise Piguet, Fanny Collaud, Giuseppe Ronzitti, Satoru Tada, Philippe Hantraye, Federico Mingozzi, Frédéric Ducongé, and Nathalie Cartier

Supplementary Results and Figure legends

Supplementary figure 1. Biodistribution of exo-AAV6-9-GFP. (A) Graph represents vector copy number into the cortex, hippocampus and striatum of adult mice transduced with std-AAV9-GFP or exo-AAV9-GFP. Data are presented as mean ± SEM. A Student t test was employed for comparisons between the two groups.

Supplementary figure 2. Detection of GFP fluorescence in vivo probe-based confocal laser endomicroscopy system between exo-AAV6-GFP and exo-AAV9-GFP. (A) Graph represents the mean between exo-AAV6 compared to exo-AAV9 of the relative fluorescence units (RFU) detected over the time into contralateral hemisphere by the 10,000 optical fibers per individual video frame. Exo-AAV6 and exo-AAV9 ($n = 8$; interaction: $F_{2,6} = 0.2095$, $p = 0.8167$; time: $F_{2,6} = 2.387$, $p = 0.1727$; spreading-contralateral: $F_{1,6} = 13.46$, $p = 0.0105$). *$p < 0.05$. Data are presented as mean ± SEM.

Supplementary figure 3. Std-AAV9 and exo-AAV9-GFP promoted GFP expression and does not trigger major glial responses in the mouse hippocampus. We assesses the effects of std-AAV9 and exo-AAV9 expression on the activation of microglial marker Iba1. We performed an immunostaining between GFP and Iba1 in brain slices containing the hippocampus of adult mice transduced with std-AAV9-GFP or exo-AAV9-GFP. The analysis ($n = 12$) by two-way-ANOVA with Tukey *post hoc test.* of Iba1 immunofluorescence of mice transduced with either std-AAV9-GFP or exo-AAV9-GFP, did not reveal statistical significant differences in the levels of this protein highlighting the safety of AAVs as a shuttle for expression of transgenes in the mouse brain. Images by Leica SP8 confocal microscope at 40x magnification. Scale bar = 50µm.

Supplementary figure 4. Quantification of GFP-positive cells between exosome-enveloped adeno-associated virus and unassociated construction following unilateral injection. (A) Graphs rapresent the quantification of GFP positive cells visible into the contralateral hemishere between std-AAV6 and exo-AAV6. CA1 ($n = 3$; $p = 0.0089$); CA2 ($n = 3$; $p = 0.0017$); CA3 ($n = 3$; $p = 0.0009$) and DG ($n = 3$; $p = 0.0051$). **(B)** Graphs rapresent the quantification of GFP positive cells visible into the contralateral hemishere between std-AAV9 and exo-AAV9. CA1 ($n = 3$; $p = 0.0058$); CA2 ($n = 3$; $p = 0.0213$); CA3 ($n = 3$; $p = 0.0279$) and DG ($n = 3$; $p = 0.0255$). *$p < 0.05$, **$p < 0.01$, ***$p < 0.001$. Data were analyzed by two-way-ANOVA with Tukey *post-hoc test.*, and expressed as percentage of GFP^{+} cells. Data are presented as mean ± SEM.

Video 1. Confocal laser endoscopy tracking of fluorescently tagged cells. Video recording of GFP positive cells in the ipsilateral and contralateral hemisphere of adult mice transduced with std-AAV6-GFP. Scale bar = 20 µm.

Video 2. Confocal laser endoscopy tracking of fluorescently tagged cells. Video recording of GFP positive cells in the ipsilateral and contralateral hemisphere of adult mice transduced with exo-AAV6-GFP. Scale bar = 20 µm.

Video 3. Confocal laser endoscopy tracking of fluorescently tagged cells. Video recording of GFP positive cells in the ipsilateral and contralateral hemisphere of adult mice transduced with std-AAV9-GFP. Scale bar = 20 µm.

Video 4. Confocal laser endoscopy tracking of fluorescently tagged cells. Video recording of GFP positive cells in the ipsilateral and contralateral hemisphere of adult mice transduced with exo-AAV9-GFP. Scale bar = 20 μm.

Supplementary figure 1

A

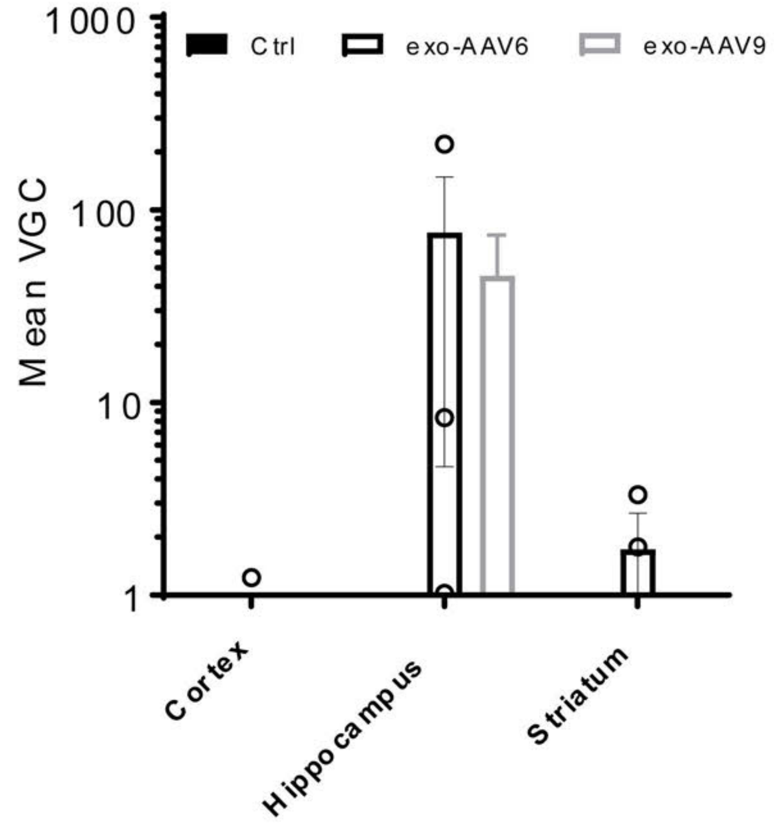

Supplementary figure 2

A

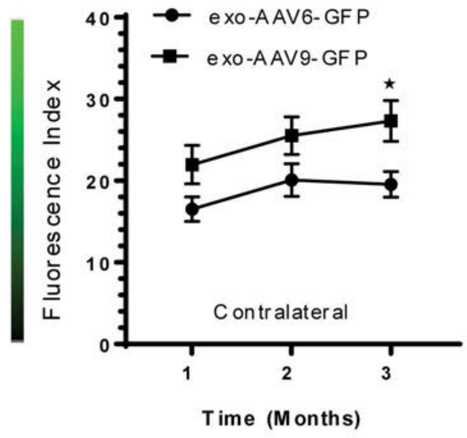

Supplementary figure 3

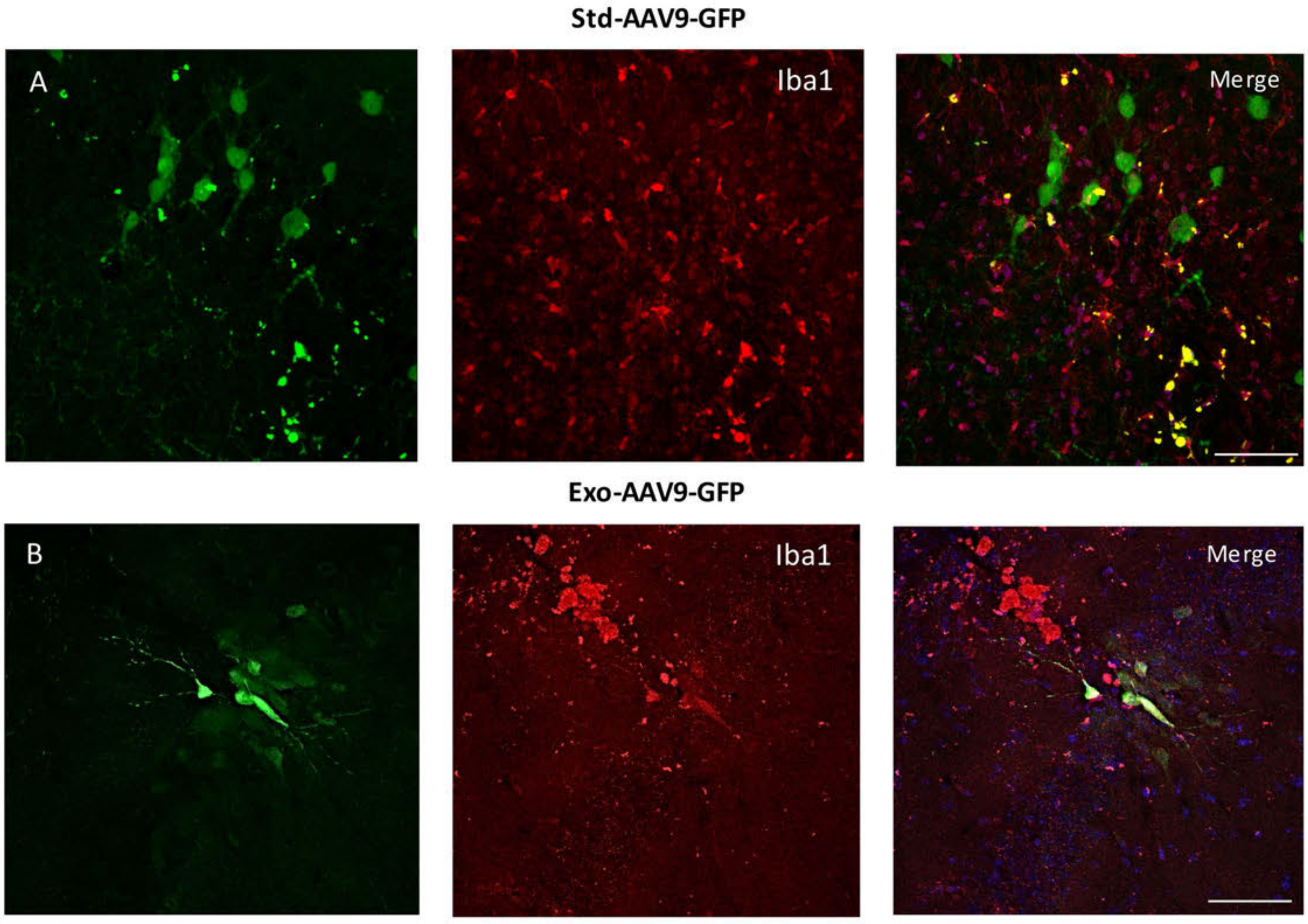

Supplementary figure 4

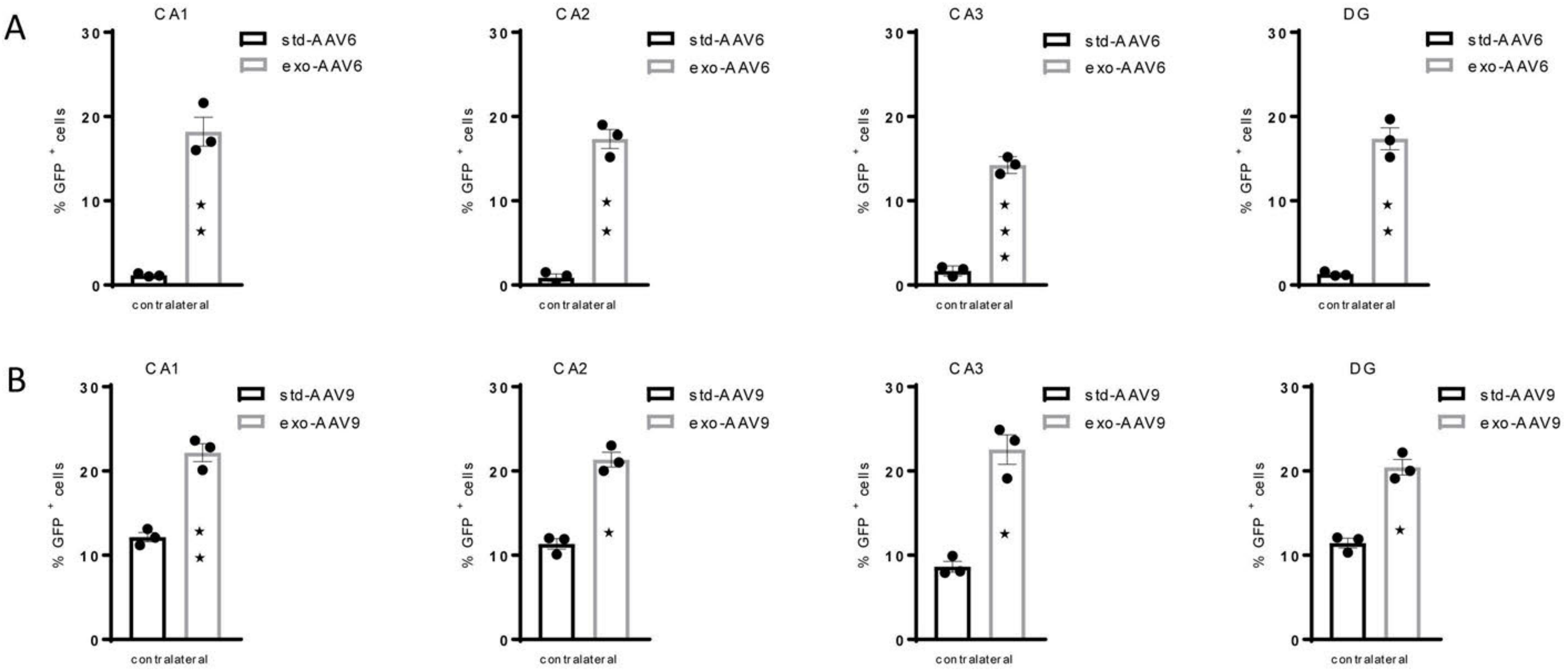

Supplement: Document S1. Figures S1–S4 [file mmc1.pdf]
